# Soluble Vascular Cell Adhesion Molecule-1 (VCAM-1) as a Biomarker in the Mouse Model of Experimental Autoimmune Myocarditis (EAM)

**DOI:** 10.1371/journal.pone.0158299

**Published:** 2016-08-08

**Authors:** U. Grabmaier, G. Kania, J. Kreiner, J. Grabmeier, A. Uhl, B. C. Huber, K. Lackermair, N. Herbach, A. Todica, U. Eriksson, L. T. Weckbach, S. Brunner

**Affiliations:** 1 Medical Department I, Ludwig-Maximilians-University, Munich, Germany; 2 Research of Systemic Autoimmune Diseases, Division of Rheumatology, University Hospital of Zurich, Zurich, Switzerland; 3 Walter Brendel Centre of Experimental Medicine, Ludwig-Maximilians-University, Munich, Germany; 4 Institute of Veterinary Pathology, Ludwig-Maximilians-University, Munich, Germany; 5 Department of Nuclear Medicine, Ludwig-Maximilians-University, Munich, Germany; 6 Cardioimmunology, Center of Molecular Cardiology, University of Zurich, Zurich, Switzerland; Monash University, AUSTRALIA

## Abstract

Vascular cell adhesion molecule-1 (VCAM-1) is strongly upregulated in hearts of mice with coxsackie virus-induced as well as in patients with viral infection-triggered dilated cardiomyopathy. Nevertheless, the role of its soluble form as a biomarker in inflammatory heart diseases remains unclear. Therefore, we investigated whether plasma levels of soluble VCAM-1 (sVCAM-1) directly correlated with disease activity and progression of cardiac dysfunction in the mouse model of experimental autoimmune myocarditis (EAM). EAM was induced by immunization of *BALB/c* mice with heart-specific myosin-alpha heavy chain peptide together with complete Freund`s adjuvant. ELISA revealed strong expression of cardiac VCAM-1 (cVCAM-1) throughout the course of EAM in immunized mice compared to control animals. Furthermore, sVCAM-1 was elevated in the plasma of immunized compared to control mice at acute and chronic stages of the disease. sVCAM-1 did not correlate with the degree of acute cardiac inflammation analyzed by histology or cardiac cytokine expression investigated by ELISA. Nevertheless, heart to body weight ratio correlated significantly with sVCAM-1 at chronic stages of EAM. Cardiac systolic dysfunction studied with positron emission tomography indicated a weak relationship with sVCAM-1 at the chronic stage of the disease. Our data provide evidence that plasma levels of sVCAM-1 are elevated throughout all stages of the disease but showed no strong correlation with the severity of EAM.

## Introduction

Myocarditis denotes cardiac inflammation, which is often triggered by viral pathogens such as adeno-, herpes-, coxsackie- or enterovirus.[1, 2] Despite the fact that myocarditis is self-limiting in up to 40%,[3] ongoing autoimmune processes and/or persistent viral infections can lead to chronic inflammation with subsequent dilation and dysfunction of the heart.[4] Although substantial efforts have been made to understand the pathophysiologic mechanisms of acute and chronic myocarditis, no established biomarker has been defined to reliably reveal the diagnosis and/or depict the severity of the disease at various stages.

Vascular cell adhesion molecule-1 (VCAM-1) is predominantly expressed on activated endothelia during inflammation as a ligand for α_1_β_4_ integrins, mediating recruitment of leukocytes.[5] Its soluble form, referred to as sVCAM-1, has been shown to correlate with disease activity in various entities including rheumatoid arthritis (RA)[6] or systemic lupus erythematosus (SLE)[7, 8] and predicts unfavourable outcome in patients with coronary artery disease (CAD).[9] We previously described VCAM-1 to be strongly upregulated in the hearts of mice with coxsackie virus-induced cardiomyopathy. Accordingly, patients with virus-induced DCM showed high expression of cardiac VCAM-1 (cVCAM-1) when compared to patients with non-inflammatory DCM, indicating that enhanced cVCAM-1 expression suggests a possible inflammatory etiology of DCM.[10] The role of sVCAM-1 as a biomarker for disease severity has not yet been defined for inflammatory heart diseases.

In this study we investigated the plasma levels of sVCAM-1 and studied the correlation between disease activity and cardiac dysfunction in the mouse model of experimental autoimmune myocarditis (EAM).

## Material and Methods

### Immunization and Treatment Protocols

Six- to 8-week old male *BALB/c* mice (Charles River, Sulzfeld, Germany) were immunized by subcutaneous injection of 200 μg heart-specific myosin-alpha heavy chain peptide (αMyHC; Ac-RSLKLMATLFSTYASADR-OH; CASLO, Lyngby, Denmark) together with complete Freund`s adjuvant (CFA; BD BIOSCIENCES, Heidelberg, Germany) on days 0 and 7 as previously described.[11] Sham mice were immunized on days 0 and 7 with CFA only. This study was carried out in strict accordance with the recommendations in the Guide for the Care and Use of Laboratory Animals of the Regierung von Oberbayern. The protocol was approved by the Committee on the Ethics of Animal Experiments of the Regierung von Oberbayern (Permit Number: GZ 55.2-1-54-2531-75-11). Mice were closely monitored throughout the experiments. None of the animals utilized for this work needed to be euthanized due to severe illness or died prior to the experimental endpoint. As indicated below, micro-PET was conducted under isoflurane anesthesia.

### ELISA

Proteins were extracted from hearts of immunized mice and healthy controls. After digestion in 0.1% collagenase for 45 minutes, cells were lysed by ultrasonic pulse echo instrument. cVCAM-1, interleukin-6 (IL-6) and transforming growth factor-β (TGF-β) proteins were quantified using commercially available kits (VCAM-1: RayBiotech, Norcross, GA, Cat# ELM-VCAM1-001; IL-6: Cat#M6000B, TGF-β: Cat#MB100B, R&D Systems, Minneapolis, MN) according to the manufacturer’s instructions. For cVCAM-1, each value represents a pooled analysis of two hearts. sVCAM-1 (R&D Systems, Minneapolis, MN, Cat# MVC00, and RayBiotech, Norcross, GA, Cat# ELM-VCAM1-001) was quantified in plasma of immunized mice, sham immunized mice and healthy controls according to the manufacturer’s instructions. For both cardiac and soluble proteins, values represent mean of 2 consecutive measurements.

### Histology

Hearts were removed, fixed in paraffin and stained with hematoxylin/eosin to assess acute infiltration (day 21). Inflammation was evaluated semiquantitatively using the EAM severity score (0, no inflammatory, infiltrates; 1, small foci of inflammatory cells between, myocytes; 2, larger foci of 100 inflammatory cells; 3, >10% of a cross section involved; and 4, >30% of a cross section involved) as previously described.[12]

### In vivo cardiac PET imaging

PET scans were performed on day 49 using a dedicated small-animal PET scanner (Inveon Dedicated PET, Preclinical Solutions, Siemens Healthcare Molecular Imaging, Knoxville, TN) as described previously.[13] Briefly, mice were anesthetized with isoflurane (1.5%) delivered via a face mask and were scanned in prone position. Fluorodeoxyglucose F 18 (FDG) was injected at a dosage of 23+/-3 MBq in a volume of approximately 100μl via a tail vein catheter. Data were processed with the Inveon Acquisition Workplace (Siemens Medical Solutions, Knoxville, TN).

### Statistical analysis

Data are shown as mean ± standard error of mean (SEM). Data were evaluated for normal distribution by the Anderson-Darling-Test. Comparison of >2 groups was conducted using one-way ANOVA followed by Tukey`s post-hoc test.

Values of p < 0.05 were considered statistically significant. Correlations were evaluated using Spearman correlation coefficient. Data were analysed using SPSS software.

## Results

### Cardiac and soluble VCAM-1 were elevated during EAM

**Fig 1:**
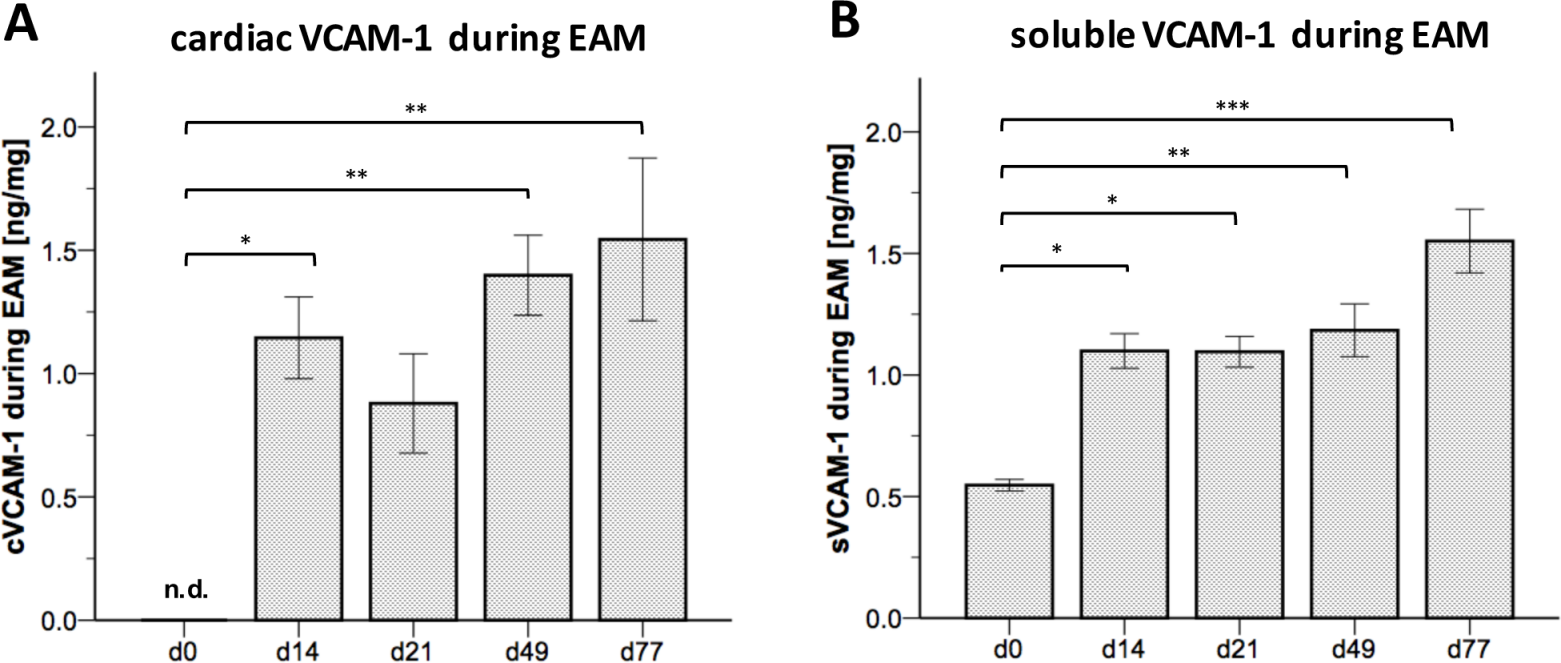
Cardiac and soluble VCAM-1 levels were elevated during EAM. **(A)** ELISA of EAM hearts revealed a distinct upregulation of cVCAM-1 at different time points, especially at the beginning of leukocyte infiltration (~ day 14) and at chronic stages of disease. **(B)** Accordingly, quantification of plasma levels of VCAM-1 showed a significant augmentation at all time points. n = 3–9 for cVCAM-1 (n = 3 for day 0, n = 9 for day 14, n = 7 for day 21, n = 6 for day 49 and day 77, each value representing a pooled analysis of 2 hearts), n = 6–12 for sVCAM-1 (n = 6 for day 0, n = 11 for day 14, n = 7 for day 21, n = 12 for day 49 and day 77). * = p < 0.05; ** = p < 0.01, *** = p < 0.001, n.d. = not detectable.

The expression of VCAM-1 was previously shown to be substantially elevated in cardiac tissue of patients with virus-induced DCM compared to patients without evidence of viral or inflammatory etiology of DCM.[10] To study the role of sVCAM-1 as a biomarker for disease severity and progression, we used the EAM model in which cardiac inflammation was induced by immunizing mice with αMyHC together with CFA. To investigate whether cVCAM-1 expression was increased in the EAM model, we performed ELISA in lysed cardiac tissue of immunized mice. cVCAM-1 expression was significantly increased during the acute phase of the disease at day 14 (p < 0.031, beginning of acute inflammation) as well as at the chronic stages at day 49 (p <0.010) and day 77 (p < 0.004) compared to baseline conditions at day 0 (Fig 1A). Comparison of baseline conditions with day 21 (peak of inflammation) almost reached significance (p = 0.166). To determine elevated sVCAM-1 plasma levels during EAM, we performed ELISA again at acute and chronic stages of the disease. In line with findings in the cardiac tissue, significantly elevated sVCAM-1 levels were observed at day 14 (p = 0.014), day 21 (p = 0.032), day 49 (p = 0.003) and day 77 (p < 0.001) compared to baseline conditions at day 0 (Fig 1B). Sham treated mice did not show elevated sVCAM-1 levels at day 21 when compared to baseline conditions at d0 (data not shown). These findings indicate elevated cVCAM-1 expression in cardiac tissue as well as elevated sVCAM-1 plasma levels during EAM.

### sVCAM-1 plasma levels did not correlate with infiltration of leukocytes or inflammatory and pro-fibrotic cytokines during EAM at peak of inflammation

**Fig 2:**
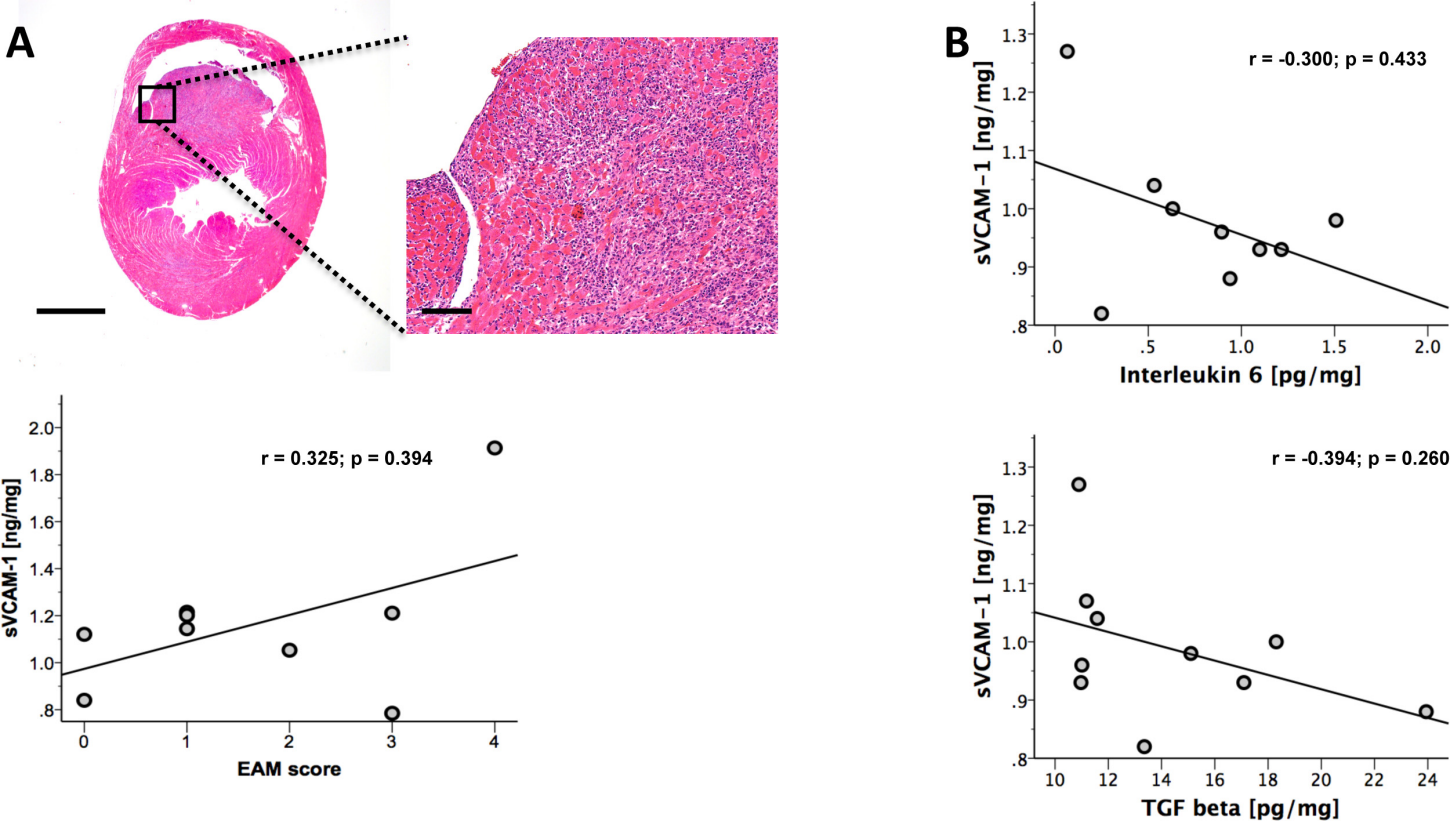
sVCAM-1 did not correlate with the grade of inflammation or pro-inflammatory or pro-fibrotic cytokine levels. **(A)** Image shows representative tissue section at day 21. sVCAM-1 did not correlate with grade of acute infiltration semi-quantitatively assessed using the EAM score. **(B)** Accordingly, the pro-inflammatory cytokine IL-6, which is critically involved in the pathogenesis of myocarditis, showed no correlation as well as the pro-fibrotic cytokine TGF-β. n = 9 for EAM score and IL-6; n = 10 for TGF-β. Bar represents 1 mm in the overview and 100 μm in the magnified image.

Next, we analyzed whether sVCAM-1 plasma levels would correlate with infiltration of leukocytes and disease severity during EAM as it was shown for SLE[7] and RA.[6] To evaluate leukocyte infiltration into the cardiac tissue during EAM, histological analysis of hearts from immunized mice at day 21 was conducted (peak of inflammation). The degree of leukocyte infiltration was determined semi-quantitatively in H&E stained cardiac cross sections using the EAM score. However, no correlation of sVCAM-1 with the severity of leukocyte infiltration was observed (r = 0.325, p = 0.394, Fig 2A). Accordingly, sVCAM-1 plasma levels did not correlate with the expression of the inflammatory marker IL-6 (r = -0.300, p = 0.433, Fig 2B) or the pro-fibrotic marker TGFβ (r = -0.394, p = 0.260, Fig 2B) analyzed in the cardiac tissue by ELISA.

### sVCAM-1 plasma levels correlated with heart/body weight ratios at different time points

**Fig 3:**
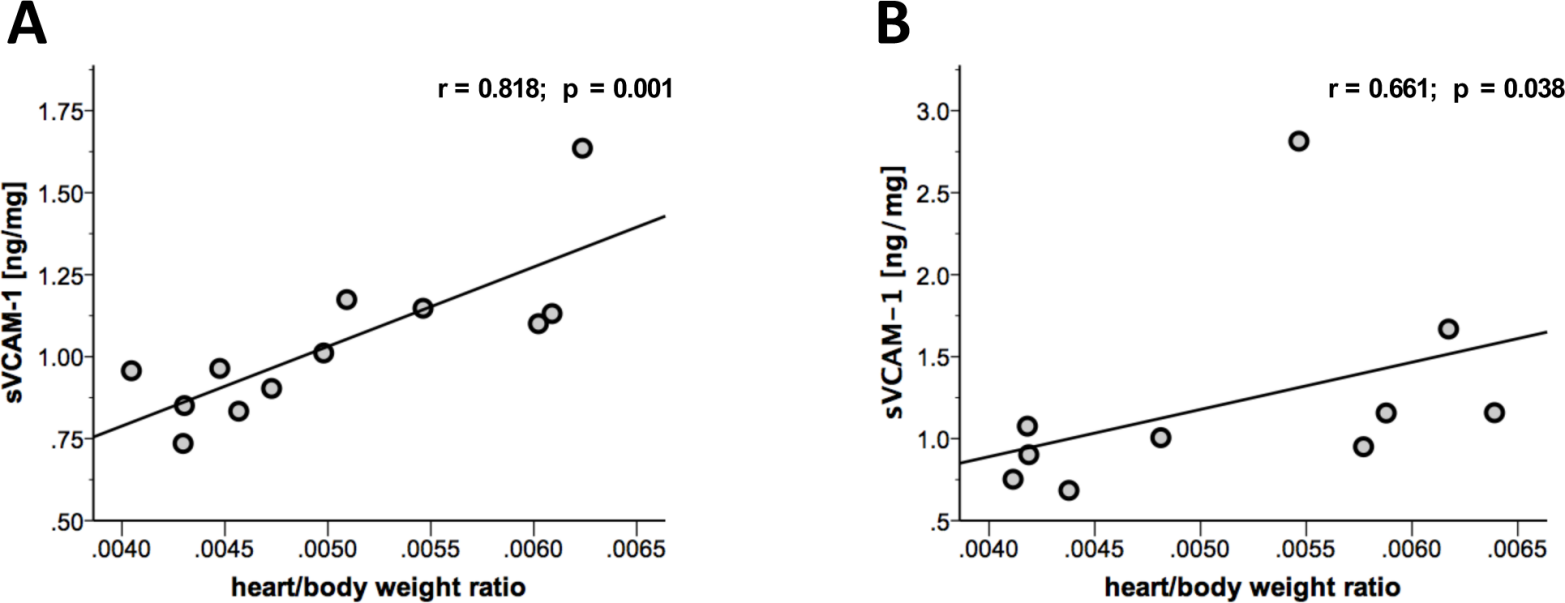
Heart/body weight ratio showed correlation with sVCAM-1 at different time points. **(A)** At day 49 (r = 0.818; p = 0.001) as well as at **(B)** day 77 (r = 0.661; p = 0.038) of disease, sVCAM-1 showed a significant correlation with heart/body weight ratio which reflects cardiac dilation at the chronic stage of disease. n = 12 for day 49, n = 10 for day 77.

Heart/body weight ratio is frequently used to depict cardiac dilation in the chronic stage of EAM.[11, 14, 15] We analyzed heart/body weight ratios in immunized mice at chronic stages of EAM (day 49 and day 77). Statistical analysis revealed correlation of sVCAM-1 with heart/body weight ratios at day 49 (r = 0.818, p = 0.001, Fig 3A) as well as day 77 (r = 0.661, p = 0.038, Fig 3B) indicating a positive correlation of sVCAM-1 with the grade of cardiac dilation.

### sVCAM-1 plasma levels showed a trend but no significant correlation with cardiac size and function at chronic stages of disease

**Fig 4:**
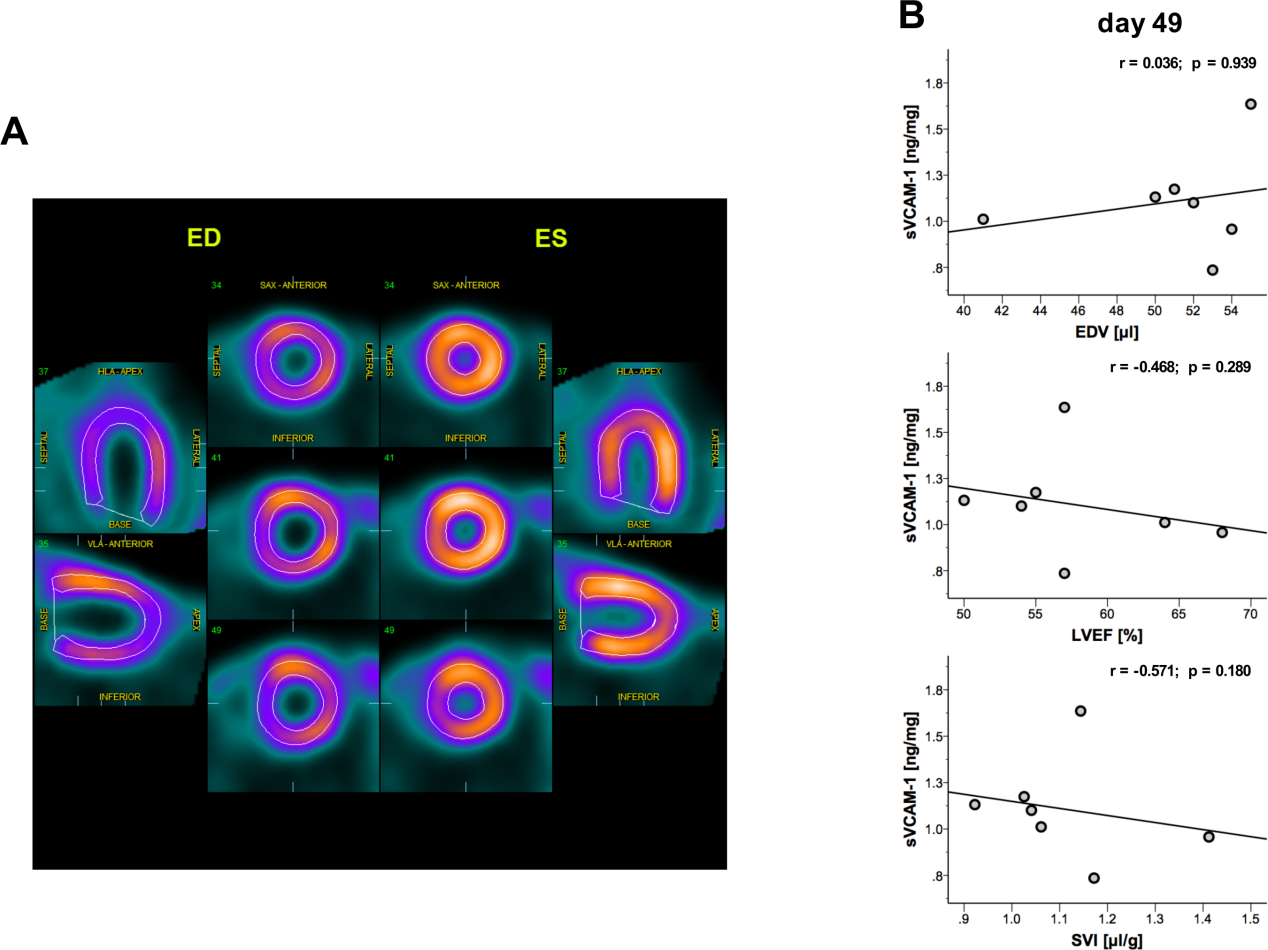
micro-PET could not verify correlation of sVCAM-1 with the grade of dilation or cardiac function. **(A)** Representative image of short and long axis view acquired with micro-PET at the end-diastolic and end-systolic period respectively. **(B)** EDV as a marker of cardiac dilation did not significantly correlate with sVCAM-1 levels. Cardiac function parameters LVEF and SVI showed a moderate yet not significant correlation at day 49. EDV = end-diastolic volume; LVEF = left ventricular ejection fraction; SVI = stroke volume index. n = 7.

To verify these findings we performed micro-PET analyzing cardiac end-diastolic volume and cardiac function parameters (Fig 4A and 4B) at day 49. As shown previously, micro-PET allows precise and reproducible evaluation of cardiac size and cardiac function in mice.[13] However, we found no correlation of sVCAM-1 with end-diastolic volume at day 49 (r = 0.036, p = 0.939, Fig 4B) and could therefore not confirm our results on cardiac dilation. Finally, ejection fraction (r = -0.468, p = 0.289, Fig 4B) and stroke volume index (r = -0.571, p = 0.180, Fig 4B) showed a moderate but not significant correlation with sVCAM-1 levels.

## Discussion

In this study we evaluated the role of sVCAM-1 as a biomarker in myocarditis in EAM, a model of myocarditis and DCMi.[16] Recent findings indicate that DCM arises from chronic ongoing inflammation due to viral persistence or chronic autoimmune processes.[17] Lately, high adiponectin levels have been shown to predict favourable outcome in patients with myocarditis.[16] Nevertheless, to date, no biomarker reliably reveals the diagnosis and/or depicts the severity of myocarditis at various stages of the disease.

We showed that in EAM, cVCAM-1 is strongly upregulated in the heart during acute and chronic stages of the disease. Our results are in line with previous findings of Eriksson et al.[18] in the EAM model and our previous findings in the animal model of coxsackie virus-induced cardiomyopathy.[10] Our results indicate that upregulation of cVCAM-1 is not restricted to viral infection of the heart but a feature of inflammation regardless of the underlying cause. According to enhanced cardiac expression, we showed for the first time that sVCAM-1 was elevated in EAM throughout the disease. To evaluate whether sVCAM-1 could function as a biomarker for the severity of EAM, we correlated sVCAM-1 levels with readouts for cardiac inflammation.

The EAM score is a widely accepted method to semi-quantitatively assess cardiac inflammation by analyzing leukocyte infiltration during the acute stage (day 21).[11, 19–21] However, no correlation could be detected between sVCAM-1 levels and grade of leukocyte infiltration assessed by EAM score at the peak of inflammation.

We next assessed the correlation of sVCAM-1 with the cardiac expression of the pro-inflammatory cytokine IL-6 and the pro-fibrotic cytokine TGF-β. IL-6 is a pro-inflammatory cytokine critically involved in the pathogenesis of myocarditis. Immunized IL-6-deficient mice showed a reduced cardiac expression of adhesion molecules intercellular cell adhesion molecule-1 (ICAM-1) and VCAM-1, reduced antibody production, and reduced activity of auto-reactive CD4+ helper cells, indicating a reduced susceptibility to EAM.[18] Furthermore, IL-6 was shown to critically influence Th-17 differentiation, which does not only affect disease initiation[22] but also the progression to dilated cardiomyopathy.[19] TGF-β has been shown to be crucial for cardiac fibrosis as it promotes differentiation of heart-infiltrating Prominin-1+ progenitor cells to cardiac fibroblasts.[11] However, sVCAM-1 did not show a correlation with cardiac IL-6 or cardiac TGF-β at the peak of inflammation.

We next examined heart/body weight ratios at the chronic stage. Increased heart/body weight ratio at the chronic stage of EAM depicts cardiac dilation.[11, 15] Interestingly, sVCAM-1 showed a statistically significant correlation with heart/body weight ratios at day 49 and day 77, indicating a correlation of sVCAM-1 with the grade of dilation at the chronic stage. To verify these findings, we investigated mice by micro-PET and evaluated end-diastolic volumes. Micro-PET is a dedicated small animal imaging device which has been previously used for the evaluation of cardiac function and dilation in a mouse model of myocardial infarction.[13] Micro-PET analysis of end diastolic volumes did not verify a correlation of sVCAM-1 with the grade of dilation in mice with EAM. We finally evaluated cardiac function parameters including LVEF and SVI. Despite a clear deterioration of EAM mice in comparison to healthy controls of a previous study,[23] cardiac function parameters did show a moderate but not significant correlation with sVCAM-1.

Our findings indicate that sVCAM-1 could serve as a marker for inflammatory dilated cardiomyopathy. However, although we observed some positive correlation of cardiac dilation and sVCAM-1, there is no clear evidence that the amount of sVCAM-1 in the plasma positively correlates with the severity of myocarditis at the acute or chronic stage.
